# Supplementary material for: Elevated Plasma Concentration of 4-Pyridone-3-carboxamide-1-β-D-ribonucleoside (4PYR) Highlights Malignancy of Renal Cell Carcinoma
Source: Int J Mol Sci. 2024 Feb 17;25(4):2359. doi: 10.3390/ijms25042359 (PMC10888534; doi:10.3390/ijms25042359)
Supplement: Supplementary file 1 [file ijms-25-02359-s001.zip › ijms-2837156-supplementary.pdf]

Elevated plasma concentration of 4-pyridone-3-carboxamide ribonucleoside (4PYR) highlights malignancy of Renal Cell Carcinoma

Supplementary Material Online

Table S1. Nicotinamide metabolites concentration in ccRCC patients with and without hypertension. Results are shown as mean ± SEM with corresponding p value by Mann–Whitney U test. NA, nicotinamide; MNA, N-methylnicotinamide; Met2PY, N-methyl-2-pyridone-5-carboxamide; Met4PY, N-methyl-4-pyridone-3-carboxamide; 4PYR, 4-pyridone-3-carboxamide-1-β-D-ribonucleoside; ccRCC, clear cell renal cell carcinoma; n, number.

**Parameter [μmol/l]**	**ccRCC patients**		
	**with hypertension (*n* = 13)**	**without hypertension (*n* = 31)**	**p value**
NA	0.28 ± 0.06	0.36 ± 0.06	0.36
Nicotinic Acid	0.08 ± 0.02	0.11 ± 0.02	0.60
MNA	0.20 ± 0.11	0.26 ± 0.05	0.65
4PYR	0.07 ± 0.009	0.08 ± 0.009	0.82
Met2PY	1.76 ± 0.28	1.56 ± 0.16	0.47
Met4PY	0.39 ± 0.05	0.35 ± 0.04	0.41

Table S2. The Glasgow prognostic scores (GPS, mGPS, HS-mGPS) in histopathological various renal carcinoma. ccRCC, clear cell renal cell carcinoma; P/Ch RCC, Papillary and Chromophobe RCC; n, number.

	GPS score			*mGPS score*			*HS-mGPS score*		
	0	***1***	***2***	***0***	***1***	***2***	***0***	***1***	***2***
ccRCC, *n*	*21*	*13*	*12*	*32*	*2*	*12*	*20*	*11*	*15*
P/Ch RCC, *n*	*6*	*3*	*1*	*9*	*0*	*1*	*9*	*0*	*1*
Oncocytoma, *n*	*4*	*4*	*0*	*7*	*1*	*0*	*4*	*3*	*1*

Table S3. Features of tumor aggressiveness in ccRCC patients at different stages and grade of development. Results are shown as mean ± SEM, * p<0.05 vs stage II by one-way ANOVA followed by Kruskal-Wallis test followed by Dunn's post hoc test, **** p < 0.0001 vs low aggressive type by unpaired t test with Welch's correction. ccRCC, clear cell renal cell carcinoma; n, number.

**Parameters**	**ccRCC *n* = 44**					
	**Low-GRADE *n* = 25**	**High-GRADE *n* = 19**	**Stage I *n* = 5**	**Stage II *n* = 10**	**Stage III *n* = 16**	**Stage IV *n* = 13**
Tumor size, cm	3.72 ± 0.35	9.15 ± 0.92 ****	7.08 ± 3.0	3.53 ± 0.79	5.86 ± 1.05	8.18 ± 0.82 *
Metastatic (no/yes)	22 / 2	5 / 13	4 /1	6 / 0	11 / 2	5 / 7
Recurrence (no/yes)	18 / 4	7 / 12	3 / 1	7 / 0	9 / 6	5 / 8
Survival (no/yes)	3 / 22	10 / 7	3 / 2	1 / 5	4 /11	5 / 7
